# HERG1 promotes esophageal squamous cell carcinoma growth and metastasis through TXNDC5 by activating the PI3K/AKT pathway

**DOI:** 10.1186/s13046-019-1284-y

**Published:** 2019-07-22

**Authors:** Hongqiang Wang, Xuchun Yang, Yan Guo, Lin Shui, Shi Li, Yifeng Bai, Yu Liu, Ming Zeng, Jianling Xia

**Affiliations:** 1 Cancer Center, Sichuan Academy of Medical Sciences and Sichuan Provincial People’s Hospital, Hospital of the University of Electronic Science and Technology of China, The Western First Round Road, Section 2#32, Chengdu, 610072 China; 2 Department of Oncology, Zhejiang Province Zhoushan Hospital, Zhoushan, 316000 China; 3 Department of Oncology, West China Hospital, West China Medical Center, Sichuan University, Chengdu, 610041 China; 4 Department of Urology, The First Affiliated Hospital of Wenzhou Medical University, Wenzhou, 325000 China

**Keywords:** Esophageal squamous cell carcinoma, Tumor progression, HERG1, TXNDC5

## Abstract

**Background:**

The human ether a-go-go-related gene 1 (HERG1) is involved in tumor progression; however, its role in esophageal squamous cell carcinoma (ESCC) is not well studied. This study investigated HERG1 function in ESCC progression and elucidated the underlying mechanisms.

**Methods:**

The prognostic value of HERG1 was determined by immunohistochemistry in ESCC biopsies. Cell growth and proliferation were analyzed by colony formation and methyl thiazolyl tetrazolium assays. Cell migration and invasion were analyzed by wound healing and Boyden transwell assays. Epithelial-mesenchymal transition (EMT) was evaluated by immunoblotting and quantitative polymerase chain reaction (qPCR). A xenograft mouse model was used to validate the tumorigenic and metastatic roles of HERG1 in vivo.

**Results:**

HERG1 expression was overall higher in ESCC tissues compared to adjacent non-tumor tissues. A retrospective analysis of 349 patients with ESCC (stages I–IV) confirmed increased HERG1 expression was associated with disease progression and higher mortality rate. The overall survival of the patients was significantly worse when their tumors displayed higher HERG1 expression. HERG1 knockdown reduced tumor growth and metastasis in athymic mice. HERG1 affected the proliferation, migration, and invasion of two ESCC cell lines (TE-1 and KYSE-30). Changes in HERG1 expression affected the expression of cell cycle- and EMT-related proteins; these effects were reversed by altering the expression of thioredoxin domain-containing protein 5 (TXNDC5), which is also associated with the clinicopathological characteristics of patients with ESCC and is relevant to HERG1 in pathological biopsies. Additionally, HERG1 expression altered phosphoinositide 3-kinase (PI3K) and AKT phosphorylation, thereby affecting TXNDC5 expression.

**Conclusions:**

HERG1 contributes to poor prognosis in patients with ESCC by promoting ESCC cell proliferation, migration, and invasion via TXNDC5 through the PI3K/AKT signaling pathway. Our findings provided novel insights into the pathology of ESCC and role of HERG1 in tumor progression, suggesting that targeting HERG1 has potential diagnostic and therapeutic value for ESCC treatment.

**Electronic supplementary material:**

The online version of this article (10.1186/s13046-019-1284-y) contains supplementary material, which is available to authorized users.

## Background

Esophageal squamous cell carcinoma (ESCC) is among the most aggressive and lethal malignancies worldwide [1, 2]. Treatment of ESCC includes chemotherapy, radiation therapy, and surgery; however, the 5-year overall survival rate of ESCC remains less than 30%, due to delayed diagnosis at an advanced stage and lack of effective targeted treatments [3]. ESCC is a multistep process involving a series of genetic and epigenetic alterations [4]; therefore, development of effective therapies for ESCC requires the identification of novel diagnostic and prognostic biomarkers, as well as adequate therapeutic targets.

The *human ether a-go-go related gene 1* (*HERG1*), located on chromosome 7, encodes for the tetrameric alpha subunit of voltage-gated potassium channel HERG1 that is found on the plasma membrane of cells [5]. During an action potential, a resurgent current that peaks in the repolarization phase is produced by this gating behavior [6]. The QT interval on the surface electrocardiogram is attributable to mutations, dysregulated expression levels, or impaired activity of HERG1 channels, which results in potentially fatal ventricular arrhythmia [7]. Meanwhile, HERG1 plays a fundamental role in the excitability of neurons, myocytes, and gland cells [8–10]. Furthermore, HERG1 has been found to be expressed in several tracts of the intestine that control gut motility by generating repolarizing currents during smooth muscle contraction. These studies demonstrated the importance of HERG1 in the physiological modulation in normal tissues [11]. However, recent studies have shown that HERG1 is closely related to the malignant phenotype of cancer cells and might be involved in the development of various cancers, including gastric cancer, breast cancer, pancreatic cancer, and osteosarcoma [12–15]. Moreover, HERG1 expression levels are critical events in the pathological conditions of several types of cancer [12, 16]. However, HERG1 expression and its associated mechanism in ESCC remain unclear.

Thioredoxins are small ubiquitous proteins with key roles in the regulation of cellular redox balance [17]. Among them, thioredoxin domain-containing protein 5 (TXNDC5) is reportedly involved in several malignancies, including cervical cancer, prostate cancer, and hepatocellular carcinoma [18–20]. Specifically, TXNDC5 promotes tumor cell differentiation, invasion, and angiogenesis [21], and its expression is positively correlated with poor survival of patients with certain tumor types [22]. However, to the best of our knowledge, the role of TXNDC5 in ESCC has not yet been investigated.

This study analyzed HERG1 expression and function, as well as its prognostic role, in ESCC, with emphasis on its functional correlation with TXNDC5, in order to evaluate the efficacy of HERG1 as a novel therapeutic target in ESCC.

## Methods

### Reagents

Phosphate-buffered saline (PBS), Roswell Park Memorial Institute medium (RPMI) 1640, and fetal bovine serum (FBS) purchased from Hyclone (Logan, UT, USA) were used. Rabbit polyclonal antibodies against human HERG1 and TXNDC5 were purchased from Abcam (Cambridge, UK), whereas those against human p21, cyclin D1, E-cadherin, vimentin, fibronectin, Janus N-terminal kinase (JNK)1/2, phosphorylated (p)-JNK1/2, p38, p-p38, phosphoinositide 3-kinase (PI3K), p-PI3K, AKT, p-AKT, Src, p-Src, and glyceraldehyde-3-phosphate dehydrogenase (GAPDH, used as the internal control) were purchased from Cell Signaling Technology (Danvers, MA, USA). Short interfering RNAs (siRNAs) that targeted TXNDC5 were obtained from GenePharma (Shanghai, China). Invitrogen (Carlsbad, CA, USA) was the supplier of TRIzol, Moloney murine leukemia virus reverse transcriptase (M-MLV-RT), and Lipofectamine 2000, whereas Roche (Penzberg, Germany) provided SYBR Green I Master kits. Unless otherwise mentioned, biochemical reagents used were from Sigma (St. Louis, MO, USA).

### Patients and tissue specimens

The study design was reviewed and approved by the Ethics Review Board of the Zhejiang Province Zhoushan Hospital (Zhoushan, Zhejiang, China) or Sichuan Provincial People’s Hospital (Chengdu, Sichuan, China). Tissue samples were collected with written informed consent from each patient. All patients had been diagnosed with ESCC upon pathological examination at the Zhejiang Province Zhoushan Hospital or Sichuan Provincial People’s Hospital from 2011 to 2014, and tumor staging was carried out according to the AJCC Cancer Staging Manual (8th edition).

### Cell culture and stable cell lines

We purchased the normal human esophageal epithelial cell line Het-1A, and the human ESCC cell lines Eca-109, EC-9706, KYSE-30, KYSE-150, KYSE-510, TE-1, and TE-13 from Foleibao Biotechnology Development Co. (Shanghai, China). Cells were grown in RPMI 1640 containing 10% FBS at 37 °C in 5% CO_2_.

Stable clones of ESCC cells overexpressing or silenced for HERG1 were generated upon transfection of plasmids containing HERG1 cDNA or short hairpin RNA (shRNA) targeting HERG1 (shHERG1) as described in a previous study [23]. Additional file 1: Tables S1 and S2 list the sequences of HERG1 cDNA and shRNAs used in this study.

For the rescue experiments, 50 nM cDNA or siRNA targeting TXNDC5 (sequences in Additional file 1: Tables S1 and S3, respectively) were transiently transfected into KYSE-30 cells stably silenced for HERG1 or into TE-1 cells overexpressing HERG1, using Lipofectamine 2000 according to the manufacturer’s instructions. Six hours after transfection, the medium was changed to RPMI 1640 supplemented with 10% FBS. The cells were then cultured for the indicated times and used for quantitative polymerase chain reaction (qPCR), cell viability, and transwell invasion assays.

### Immunohistochemistry (IHC)

IHC was conducted on formalin-fixed, paraffin-embedded tumor tissues, as described in our previous studies [23, 24]. Two independent pathologists analyzed the expression of the target protein by visualizing the brown-stained section. The staining intensity of the tumor samples was scored as follows: 0 (negative), 1 (weak), 2 (moderate), or 3 (strong). The staining extent of the tumor samples was scored according to the percentage of positive cells in the whole tissue slices: 0 (0%), 1 (1–25%), 2 (26–50%), 3 (51–75%), or 4 (76–100%). The staining intensity and staining extent scores were then added to calculate the final stain score (0–7) for each tumor tissue.

### qPCR

Total RNA was isolated from tissues or cells with TRIzol and reverse transcribed using a PrimeScript M-MLV-RT kit. A SYBR Green I Master kit was used to conduct qPCR with a LightCycler 480 system (Roche). The primer sequences used for qPCR are listed in Additional file 1: Table S4.

### Western blot analyses

Tissue specimens or cell lines were lysed using cell lysis buffer. Subsequently, 10% sodium dodecyl sulfate polyacrylamide gel electrophoresis was employed to resolve equal amounts of proteins, which were then transferred onto polyvinylidene difluoride membranes (Millipore, Billerica, MA, USA). The membranes were then blocked with 5% bovine serum albumin (BSA), incubated with the indicated primary antibodies overnight at 4 °C, washed, and incubated with the appropriate secondary antibodies for 1 h. Antibody-bound protein bands were visualized with the enhanced chemiluminescence method on X-ray films.

### Cell viability analyses

ESCC cells were seeded at an initial density of 4 × 10^3^ cells per well on 96-well plates, and the cell viability was determined using methyl thiazolyl tetrazolium (MTT) assays over 24 h to 96 h. Specifically, 20 μL of MTT solution (5 mg/mL in PBS) was added to each well, and the plates were incubated for 4 h at 37 °C. Further, 150 μL of dimethyl sulfoxide was added to each well replacing the previous medium and incubated for 10 min. The absorbance was recorded at 570 nm using a microplate spectrophotometer (Thermo Fisher Scientific, Waltham, MA, USA).

### Cell proliferation assays

A total of 1 × 10^3^ cells per well were plated in 12-well plates to carry out the cell proliferation assays. We set up three replicate wells for each group in each experiment. Each experiment was repeated three times. The gross number of cells in each well was counted at 0, 24, 48, 72, and 96 h.

### Colony formation assays

Two hundred ESCC cells were seeded onto 6-well plates, cultured for 2 weeks, and then fixed with 4% paraformaldehyde for 20 min before staining with 0.1% crystal violet for 20 min. After washing out excess dye, the colonies were observed and photographed.

### Bromodeoxyuridine (BrdU) incorporation assays

TE-1 or KYSE-30 cells were seeded onto glass coverslips (Thermo Fisher Scientific, Pittsburgh, PA, USA) and serum-starved for 48 h. Subsequently, the cells were incubated with 10 mM BrdU for 1 h, followed by staining with an anti-BrdU antibody (Upstate, Temecula, CA, USA). Cell nuclei were dyed using 4′,6-diamidino-2-phenylindole (DAPI, Invitrogen). Labeled cells were imaged using an Olympus BX51 microscope (Olympus, Tokyo, Japan).

### Cell cycle analyses via flow cytometry

To perform cell cycle analysis, TE-1 or KYSE-30 cells were collected by trypsinization and fixed in 70% ethanol at 4 °C overnight. The cells were then incubated in PBS containing 0.1% Triton-X-100 and 100 μg/mL RNase A for 30 min at 37 °C, and stained with 50 μg/mL propidium iodide (PI) in PBS for another 30 min at 37 °C. Data were acquired with a FACS Vantage flow cytometer (BD Biosciences, Franklin Lakes, NJ, USA) and analyzed with the FlowJo software (FlowJo LLC., Ashland, OR, USA).

### Apoptosis analyses via flow cytometry

For apoptosis analysis, cells were seeded in 6-well plates at a density of 5 × 10^5^ cells per well. After incubation at 37 °C and 5% CO_2_, floating and adherent cells were harvested and stained with 5 μL annexin V-FITC and 50 μg/mL PI for 30 min in the dark. Cells were then immediately analyzed by flow cytometry. The percentage of cells in the early stages of apoptosis was calculated by counting the number of cells positive for annexin V and negative for PI.

### Fluorescence staining

ESCC cells were plated on glass cover slips. They were then washed with PBS, fixed with 3.7% formaldehyde solution, permeabilized with 0.1% Triton X-100 in PBS-Tween, and blocked with 5% BSA. Next, cells were stained with rhodamine-conjugated phalloidin for 1 h at ambient temperature to detect F-actin. Finally, cells were imaged using an Olympus FluoView confocal microscope.

### Terminal deoxytransferase mediated dUTP-biotin nick end labeling (TUNEL) assays

Cell apoptosis also was examined by TUNEL assay. Cells were cultured in 6-well plates and incubated at 37 °C and 5% CO_2_. Cells were washed and then stained using the ApoBrdU DNA Fragmentation Assay Kit (Roche) according to the manufacturer’s protocol. Apoptosis was observed using confocal laser scanning microscopy (Olympus).

### Wound healing assays

Wound healing assays were implemented to evaluate the cell migration ability. Cells were seeded in 6-well plates and grown to confluency, after which, artificial wounds were created using a 200-μL pipette tip. Wound closure was observed and imaged after 24 h using an Olympus BX51 microscope.

### Transwell invasion assays

Transwell invasion assays were conducted using Boyden’s chambers (Corning Life Sciences, Lowell, MA, USA). Cells were seeded in the upper chambers of 8-mm membrane filter inserts (4 × 10^4^ cells/well) coated with Matrigel (BD Biosciences, NY, USA). The chemoattractant in the lower chambers was the medium containing 10% FBS. After incubation for 24 h at 37 °C, the cells that invaded the lower surface of the inserts, through the coated membrane, were fixed with 4% paraformaldehyde, stained with 0.1% crystal violet, photographed under an Olympus BX51 microscope, and counted.

### Xenograft model

Four-week-old athymic nude mice were injected subcutaneously in the right flank regions with either 5 × 10^6^ cells of KYSE-30/scramble, KYSE-30/shHERG1 #1, or KYSE-30/shHERG1 #2. Tumor volumes were monitored every 4 days, and tumor size was calculated using the following formula: tumor volume = width × length × (width + length) × 0.5. Mice were euthanized on day 28 after injection to isolate tumors for photographing, IHC analysis, and western blotting. Animal studies were conducted according to the protocols approved by the Sichuan Provincial People’s Hospital Ethics Review Board.

KYSE-30 cells (2 × 10^6^ cells/mouse) with or without altered HERG1 expression were intravenously injected into athymic mice in order to assess whether HERG1 affects tumor metastasis in vivo. At 35-day post-implantation, mice were administered 100 mg/kg D-luciferin (Xenogen, Alameda, CA, USA) via peritoneal injection 5 min before bioluminescent imaging (IVIS 100 Imaging System; Xenogen), and lungs were harvested for ex vivo imaging. Metastases in the lungs were then visualized and verified using hematoxylin and eosin (H&E) staining.

### Statistics

All experiments were carried out at least three times, unless otherwise indicated. The results show one representative experiment. Data represent the mean ± standard error of mean (SEM). *p* values < 0.05 were deemed to be statistically significant.

## Results

### HERG1 is highly expressed in ESCC and is linked to poor clinical outcomes

**Fig. 1:**
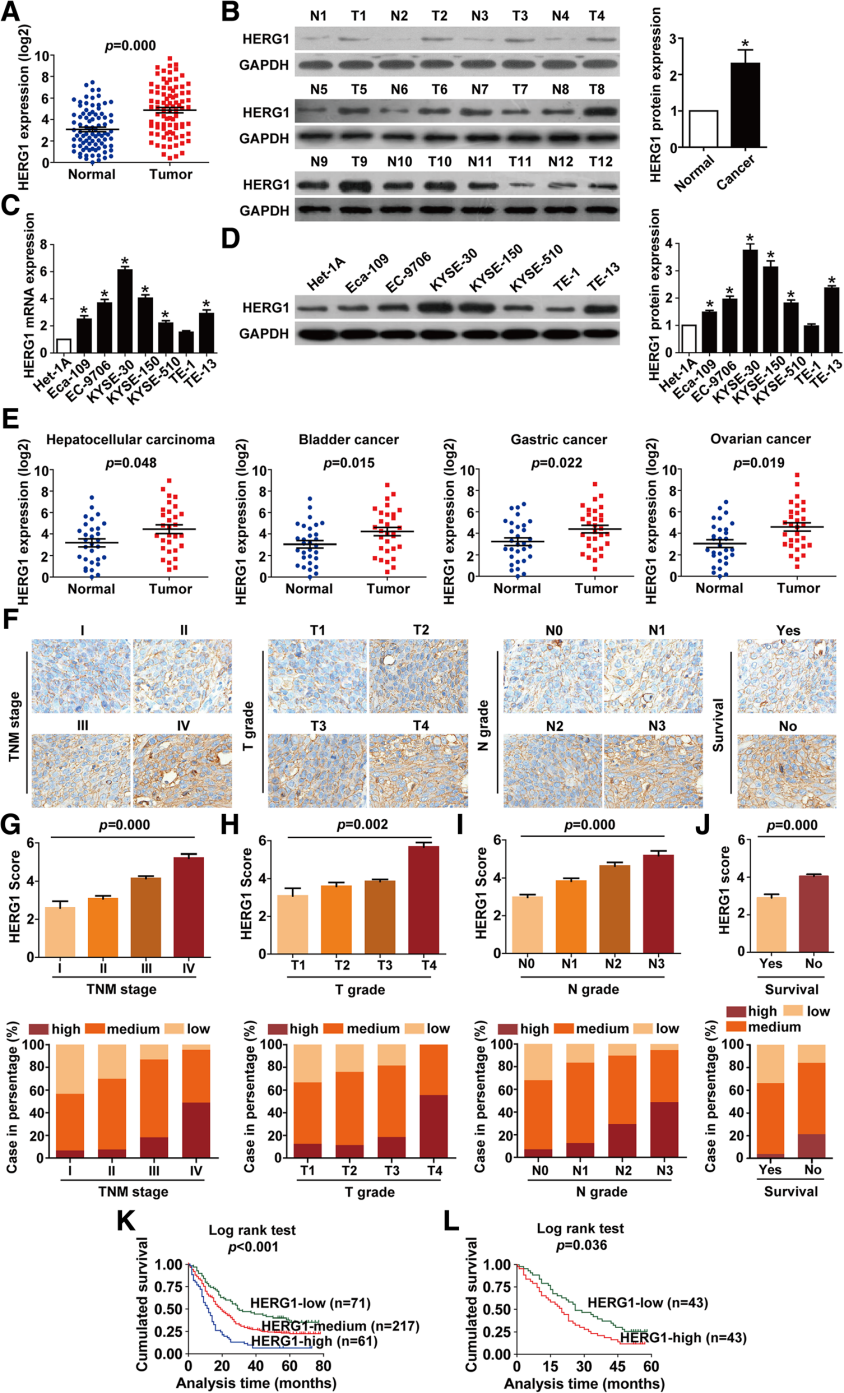
Expression of HERG1 is upregulated in ESCC tumor tissues and cell lines and correlates with ESCC clinicopathological characteristics. (**a** and **b**) Levels of HERG1 in ESCC tumor tissues and normal adjacent tissues were examined by qPCR (**a**; *n* = 86) and western blotting (**b**; *n* = 12). (**c** and **d**) qPCR (**c**) and western blotting (**d**) were used to analyze levels of HERG1 in the indicated ESCC cell lines and normal esophageal epithelial cells; *n* = 3. (**e**) HERG1 mRNA was frequently upregulated in hepatocellular carcinoma, bladder cancer, gastric cancer, and ovarian cancer (*n* = 30). Immunohistochemical staining of 349 primary ESCC specimens for HERG1 protein was performed using paraffin-embedded ESCC tissue specimens. (**f**) Representative images of HERG1 staining according to the TNM stage, T grade, N grade, and survival status of the patients (magnification, 200×). (**g**–**j**) HERG1 staining scores and frequency of high, medium, and low protein expression for TNM stages (**g**), T grades (**h**), N grades (**i)**, and in relation to the survival of the patients (**j**); *n* = 349. (**k** and **l**) Kaplan–Meier analysis of the survival rates of patients with ESCC in relation to HERG1 protein (**k**; *n* = 349) and mRNA (**l**; *n* = 86) expression. *: *p* < 0.05

We first detected the mRNA expression of HERG1 in tissues by qPCR and found that HERG1 mRNA levels were elevated in ESCC tissues compared to adjacent normal tissues in 69.8% (60/86) of the patients (Fig. 1a). Western blotting results also showed that HERG1 expression was significantly higher in 83.3% (10/12) of examined ESCC tissue samples than in the adjacent normal tissues (Fig. 1b). We further examined HERG1 expression in seven human ESCC cell lines (Eca-109, EC-9706, KYSE-30, KYSE-150, KYSE-510, TE-1, and TE-13) and a normal esophageal epithelial cell line (Het-1A). We also found that, overall, HERG1 expression was higher at both mRNA (Fig. 1c) and protein levels (Fig. 1d) in ESCC cell lines compared with Het-1A cells. As the TE-1 cell line exhibited the lowest HERG1 levels in the tested ESCC cell lines, we chose this cell line for subsequent overexpression experiments. Additionally, because the KYSE-30 cell line exhibited the highest HERG1 expression among the tested ESCC cell lines, we chose this cell line for subsequent knockdown experiments. Moreover, HERG1 was found to be significantly upregulated in other types of cancer, such as hepatocellular carcinoma, bladder cancer, gastric cancer, and ovarian cancer (Fig. 1e).

IHC staining was then used to examine HERG1 expression in 349 patients. Patients were classified as exhibiting low (score 0–2), medium (score 3–5), or high (score 6–7) HERG1 expression levels based on the scoring system described in the Materials and Methods section. Representative specimens of each TNM stage, T grade, N grade, and survival are shown in Fig. 1f. Notably, HERG1 score and the frequency of elevated protein level were significantly higher in samples at more advanced TNM stages (*p* = 0.000), T grades (*p* = 0.002), N grades (*p* = 0.000), and those from patients who were dead at the completion of the follow-up (*p* = 0.000) (Fig. 1g–j).

Importantly, based on clinical follow-up data, higher HERG1 levels were indicative of poorer prognosis (Additional file 1: Table S5). Compared with the patients with medium or low HERG1 expression, those with high HERG1 expression presented lower overall survival rates (Fig. 1k). Moreover, according to HERG1 mRNA expression levels, patients with higher levels of HERG1 expression exhibited a worse prognosis (Fig. 1l).

### HERG1 promotes the proliferation of ESCC cells

**Fig. 2:**
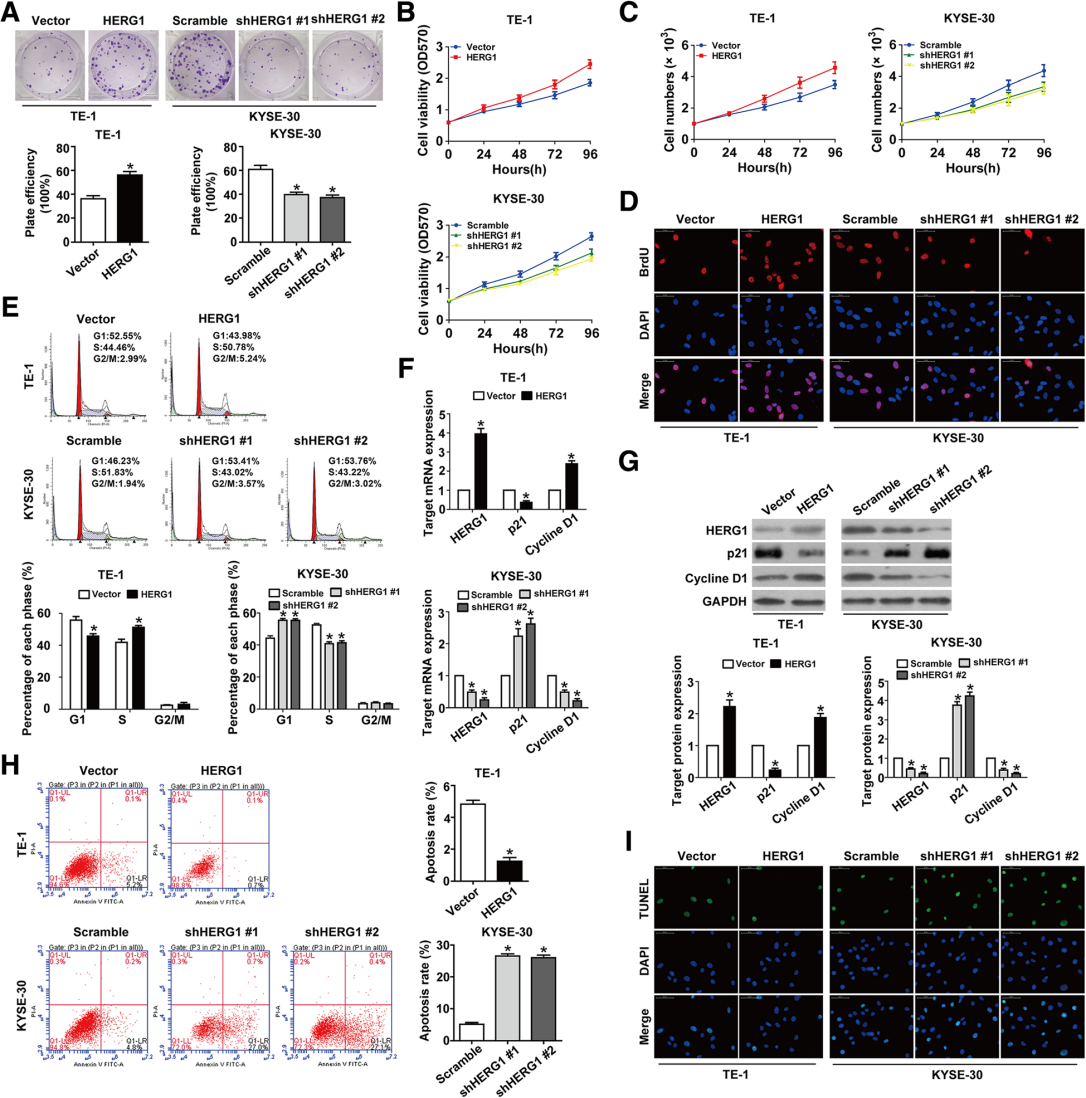
HERG1 stimulates proliferation of ESCC cells. **a** Colony formation was enhanced in HERG1-overexpressing TE-1 cells and reduced in HERG1-silenced KYSE-30 cells (*n* = 3). **b** In MTT assays, cell viability was increased by HERG1 overexpression and decreased by HERG1 silencing (*n* = 3). **c** Equal cell densities (1 × 10^3^) with different HERG1 expression were plated into 12-well plates, and the cells were counted at 0, 24, 48, 72, and 96 h (n = 3). **d** BrdU assays showed increased DNA synthesis in HERG1-overexpressing TE-1 cells, and decreased DNA synthesis in HERG1-silenced KYSE-30 cells (*n* = 3). **e** HERG1-overexpressing cells proceeded faster than control cells into the S phase, whereas HERG1-silenced cells were arrested in the G1 phase (*n* = 3). (**f** and **g**) mRNA and protein levels of cell cycle-related molecules p21 and cyclin D1 in different conditions were analyzed by qPCR (**f**) and western blotting (**g**); *n* = 3. (**h** and **i**) Cell apoptosis was evaluated by flow cytometry (**h**) and TUNEL assays (**i**) in TE-1 cells and KYSE-30 cells with different HERG1 expression (*n* = 3). *: *p* < 0.05

ESCC cell growth and proliferation were promoted by ectopic HERG1 expression and inhibited by HERG1 silencing, as determined by colony formation and MTT assays (Fig. 2a and b). Equal concentrations of cells were also seeded into 12-well plates, and HERG1 overexpression/knockdown increased/decreased the number of ESCC cells compared to that in the control group (Fig. 2c). These findings were further confirmed in BrdU assays (Fig. 2d). Moreover, HERG1 overexpression and knockdown prevented and caused, respectively, a growth arrest in the G1 phase of the cell cycle (Fig. 2e). qPCR and western blot analyses were then conducted to quantify expression of cell cycle-related molecules. HERG1 overexpression induced downregulation of p21 and upregulation of cyclin D1 in ESCC cells, whereas HERG1 knockdown induced upregulation of p21 and downregulation of cyclin D1 (Fig. 2f and g), suggesting that HERG1 is involved in ESCC cell cycle progression. Meanwhile, flow cytometry analyses using annexin V/PI staining and TUNEL assays demonstrated that upregulating HERG1 in TE-1 cells inhibited cell apoptosis, whereas cell apoptosis was induced by the knockdown of HERG1 in KYSE-30 cells (Fig. 2h and i).

### HERG1 enhances the migration and invasion of ESCC cells

**Fig. 3:**
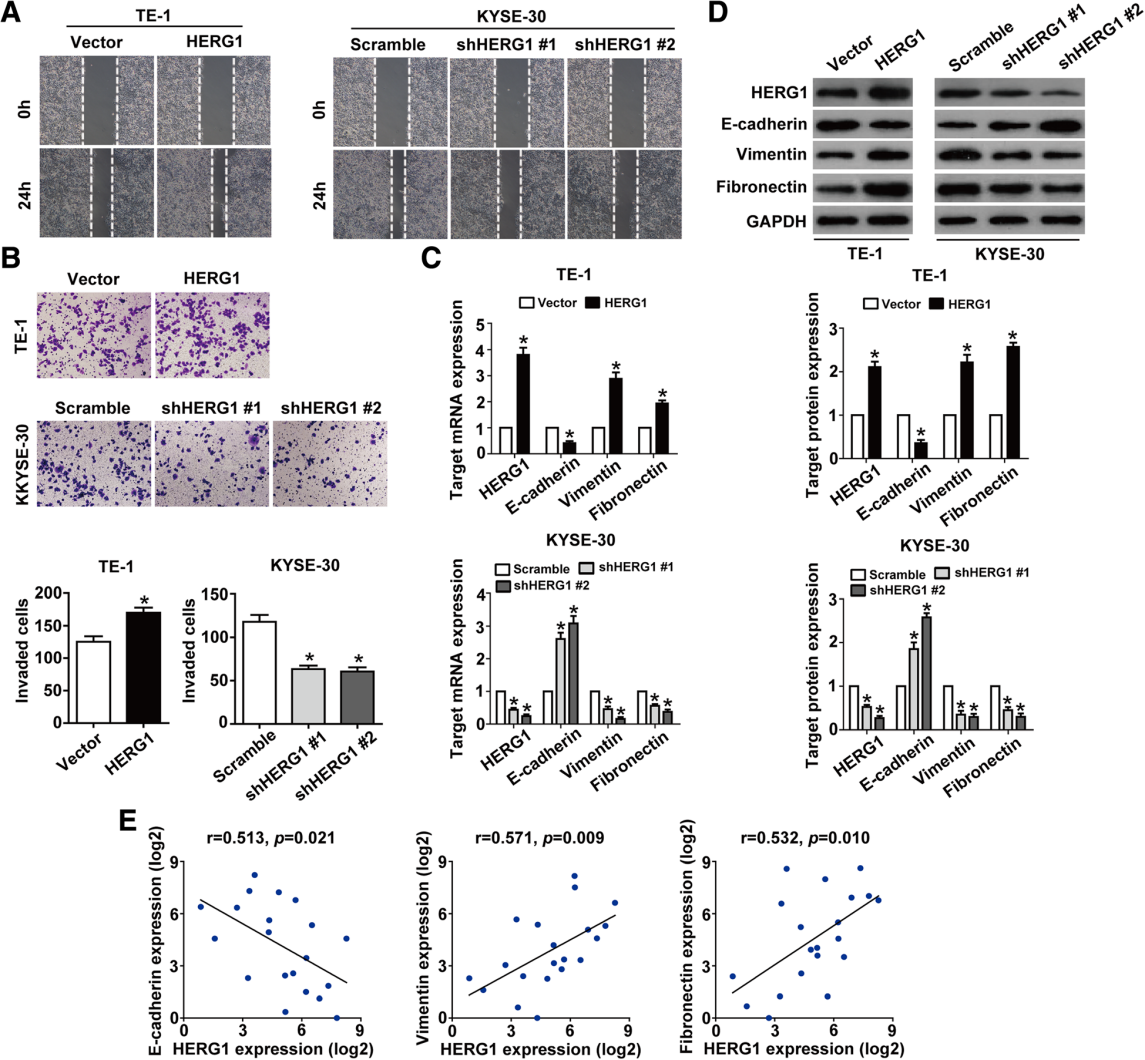
HERG1 enhances migration and invasion of TE-1 and KYSE-30 cells. **a** Cell migration was assessed in wound healing assays. After 24 h, the wound was nearly closed upon HERG1 overexpression in TE-1 cells and was wider following HERG1 knockdown in KYSE-30 cells (*n* = 3). **b** In Boyden chamber invasion transwell assays, invasiveness was quantified by counting the cells invading through Matrigel into the bottom chamber (*n* = 3). **c** and **d** mRNA and protein levels of HERG1, E-cadherin, vimentin, and fibronectin in the different treatment groups were analyzed by qPCR (**c**) and western blotting (**d**); *n* = 3. (**e**) qPCR results showed that the expression of HERG1 was significantly correlated with that of the EMT markers, E-cadherin, vimentin, and fibronectin in samples of patients with ESCC; *n* = 20. *: *p* < 0.05

Wound healing assays showed that HERG1 overexpression substantially accelerated wound closure, whereas HERG1 knockdown had opposite effects (Fig. 3a). In addition, in Boyden transwell invasion assays, we found that the number of invading cells was significantly higher in HERG1-overexpressing cells compared to control (transfected with the empty vector) TE-1 cells and significantly lower in HERG1-silenced cells compared to control (scramble shRNA-transfected) KYSE-30 cells (Fig. 3b). Interestingly, the shape of TE-1 cells transformed from round to spindle-like mesenchymal appearance of F-actin fibers after upregulation of HERG1, whereas downregulation of HERG1 in KYSE-30 cells showed the opposite result (Additional file 1: Figure S1).

We then examined the expression of E-cadherin, vimentin, and fibronectin, which are important epithelial-mesenchymal transition (EMT) markers that are involved in cell migration and invasion, in relation to HERG1 levels. qPCR and western blotting analyses showed that in HERG1-overexpressing TE-1 cells, levels of vimentin and fibronectin were significantly higher, whereas E-cadherin expression levels were significantly lower than in control cells. However, downregulation of HERG1 in KYSE-30 cells had an opposite effect (Fig. 3c and d). In addition, significant correlation was found between the expression of HERG1 and that of E-cadherin (r = 0.513, *p* = 0.021), vimentin (r = 0.571, *p* = 0.009), and fibronectin (r = 0.532, *p* = 0.010) (Fig. 3e).

### HERG1 induces ESCC progression by targeting TXNDC5

**Fig. 4:**
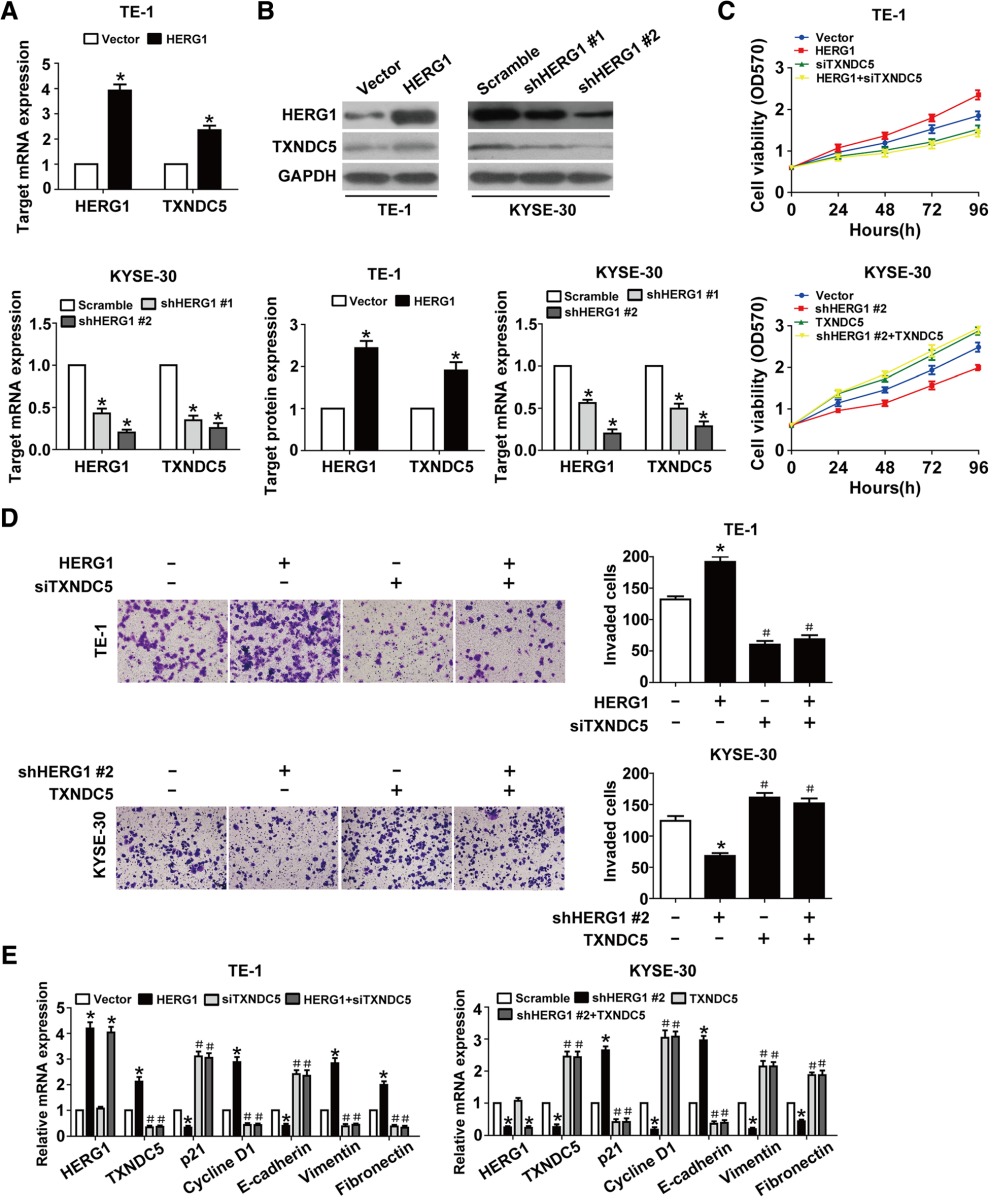
TXNDC5 is required for HERG1-mediated proliferation, invasion, and EMT in ESCC cells. (**a** and **b**) TE-1 cells transfected with a HERG1-overexpressing or control plasmid, and KYSE-30 cells transfected with HERG1 or scramble shRNA were subjected to qPCR (**a**) and western blot analyses (**b**) for the detection of HERG1 and TXNDC5 expression (*n* = 3). (**c** and **d**) MTT assays and Boyden chamber invasion transwell assays were used to assess cell proliferation (**c**) and invasion (**d**) in TE-1 cells overexpressing HERG1 and/or silenced for TXNDC5, and KYSE-30 cells silenced for HERG1 and/or overexpressing TXNDC5 (*n* = 3). (**e**) TE-1 and KYSE-30 cells in different treatment groups were subjected to qPCR for the detection of HERG1, TXNDC5, p21, cyclin D1, E-cadherin, vimentin, and fibronectin expression (*n* = 3). *: *p* < 0.05, #: *p* < 0.05 vs. HERG1 or HERG1 shRNA

Interestingly, TXNDC5 mRNA and protein levels in ESCC cells were clearly increased upon HERG1 overexpression and decreased upon HERG1 knockdown (Fig. 4a and b). We silenced TXNDC5 in HERG1-overexpressing TE-1 cells or overexpressed TXNDC5 in HERG1-silenced KYSE-30 cells in order to explore the interplay between TXNDC5 and HERG1 in the proliferation and invasion of ESCC cells. MTT and Boyden chamber invasion assays indicated that the downregulation and upregulation of TXNDC5 counteracted the effects induced by HERG1 overexpression and downregulation, respectively, on ESCC cell proliferation and invasion (Fig. 4c and d). We obtained similar results by analyzing the levels of cell cycle-related and EMT markers upon simultaneous TXNDC5 knockdown and HERG1 overexpression or TXNDC5 overexpression and HERG1 silencing (Fig. 4e).

### TXNDC5 expression is correlated with that of HERG1 and is linked to poor clinical outcomes in patients with ESCC

**Fig. 5:**
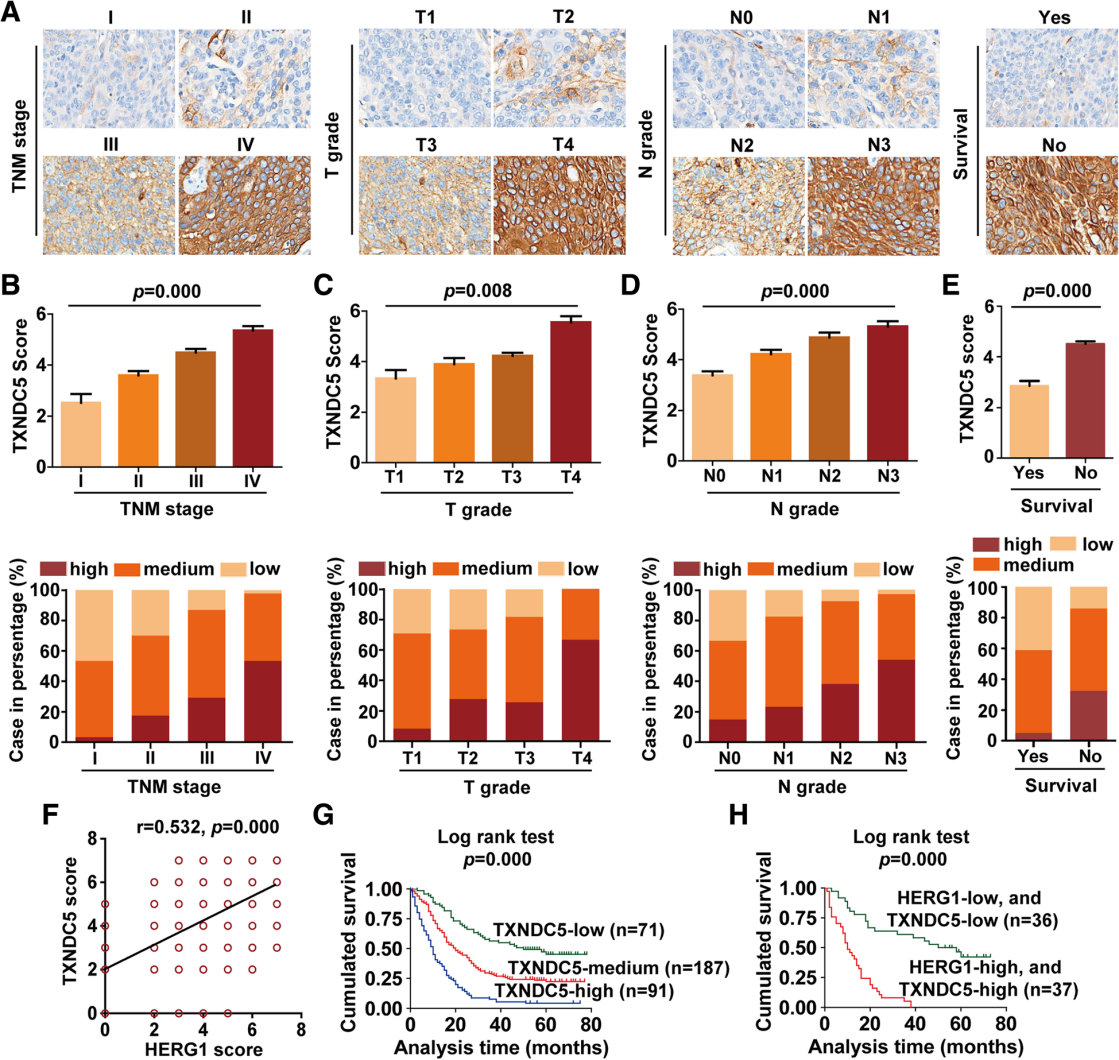
TXNDC5 expression in patients with ESCC, and its correlation with HERG1 and the clinicopathological characteristics of ESCC. Immunohistochemical staining of 349 primary ESCC specimens for TXNDC5 was performed using paraffin-embedded ESCC tissue specimens. **a** Representative images of TXNDC5 staining according to the TNM stage, T grade, N grade, and survival status of the patients (magnification, 200×). **b**–**e** TXNDC5 staining scores and frequency of high, medium, and low protein expression for TNM stages (**b**), T grades (**c**), N grades (**d**), and in relation to the survival of the patients (**e**); *n* = 349. (**f**) Positive correlation between the protein levels of HERG1 and TXNDC5 in the 349 ESCC specimens tested. (**g**) Kaplan–Meier analysis of the survival rates of patients with ESCC in relation to TXNDC5 protein expression (*n* = 349). (**h**) The log-rank test was used to assess the survival of patients with ESCC in relation to both high HERG1 and TXNDC5 expression, or both low HERG1 and TXNDC5 expression (*n* = 73). *: *p* < 0.05

IHC staining was performed to analyze TXNDC5 expression in the same 349 patients described above. The staining results of representative specimens of each TNM stage, T grade, N grade, and survival are shown in Fig. 5a. We noted that TXNDC5 score and the frequency of high protein expression were significantly higher in samples with more advanced TNM stages (*p* = 0.000), T grades (*p* = 0.008), N grades (*p* = 0.000), and those from patients who were dead at the completion of follow-up (*p* = 0.000) (Fig. 5b–e). Notably, the results of these analyses also indicated a positive correlation between HERG1 and TXNDC5 expression (Fig. 5f; r = 0.532, *p* = 0.000).

Furthermore, higher TXNDC5 levels were associated with poorer prognosis of the patients (Additional file 1: Table S6), and patients with high TXNDC5 expression showed lower overall survival rates (Fig. 5g). Notably, the overall survival rates of the patients were significantly decreased in patients with both high HERG1 and TXNDC5 expression than in patients with both low HERG1 and TXNDC5 expression (Fig. 5h).

### The PI3K/AKT signaling pathway participates in HERG1-mediated upregulation of TXNDC5

**Fig. 6:**
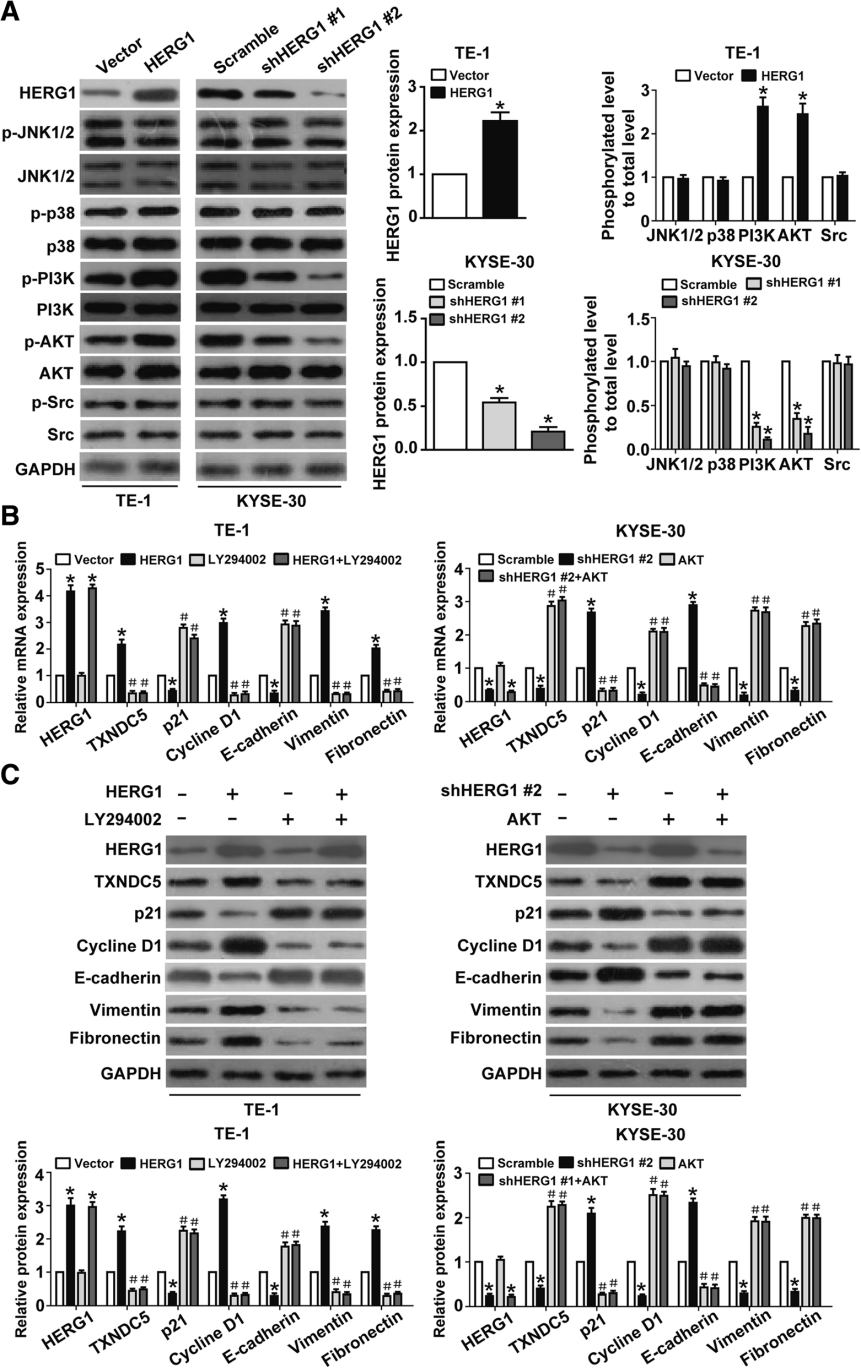
PI3K/AKT signaling pathway plays an important role in HERG1-dependent TXNDC5 upregulation. **a** HERG1-overexpressing TE-1 cells and HERG1-knockdown KYSE-30 cells were analyzed by western blotting to assess the expression of HERG1 and accumulation of JNK1/2, p38, PI3K, AKT, and Src, as well as their phosphorylated forms (*n* = 3). (**b** and **c**) HERG1, TXNDC5, p21, cyclin D1, E-cadherin, vimentin, and fibronectin expression in TE-1 cells administered the PI3K/AKT inhibitor LY294002 for 24 h in the presence or absence of HERG1 overexpression according to qPCR (**b**) and western blot analyses (**c**). HERG1, TXNDC5, p21, cyclin D1, E-cadherin, vimentin, and fibronectin expression in KYSE-30 cells transfected with HERG1 shRNA and/or AKT cDNA according to qPCR (**b**) and western blot analyses (**c**) (*n* = 3)

To elucidate the signaling pathway involved in HERG1-dependent TXNDC5 upregulation in ESCC cells, we investigated the effect of HERG1 on the phosphorylation of signaling molecules, such as JNK1/2, p38, AKT, and Src. Although we observed an increase in the level of the phosphorylated (active) form of PI3K and AKT upon HERG1 overexpression in TE-1 cells, there was a decrease in PI3K and AKT phosphorylation upon HERG1 knockdown in KYSE-30 cells. Additionally, no changes were observed in the phosphorylation of JNK1/2, p38, and Src upon altering HERG1 expression (Fig. 6a). TE-1 cells treated with LY294002 (a PI3K/AKT inhibitor) for 24 h in combination with or without HERG1 overexpression exhibited significant decreases in TXNDC5, cyclin D1, vimentin, and fibronectin expression and increases in p21 and E-cadherin mRNA levels (Fig. 6b), whereas KYSE-30 cells exhibiting AKT overexpression in the presence or absence of HERG1 knockdown displayed significant increases in TXNDC5, cyclin D1, vimentin, and fibronectin expression and decreases in p21 and E-cadherin mRNA levels. These findings were confirmed by western blot analyses (Fig. 6c).

### HERG1 accelerates tumor growth and metastasis in vivo

**Fig. 7:**
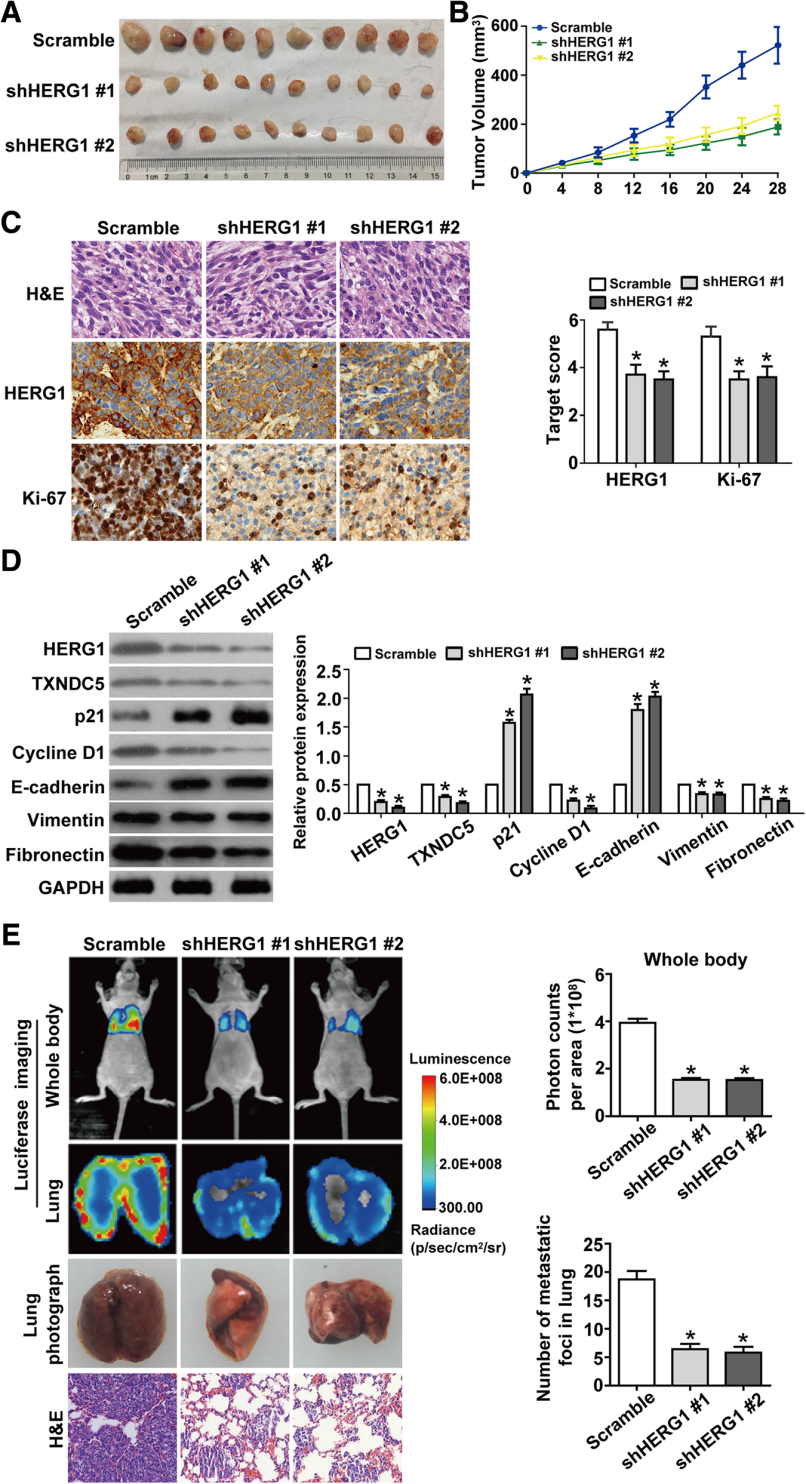
HERG1 boosts tumor growth and metastasis in athymic mice. **a** KYSE-30/scramble, KYSE-30/shHERG1 #1, or KYSE-30/shHERG1 #2 tumor cells were subcutaneously injected into the back of female athymic mice to evaluate tumorigenesis. Twenty-eight days post-implantation, the volumes of the generated tumors were compared among the three groups (*n* = 10). **b** Tumor volumes were measured in the three experimental groups over 28 days (*n* = 10). **c** Representative images from immunohistochemical analysis of tumor samples stained with H&E and with a HERG1 and Ki-67 antibody (*n* = 10). Magnification: 200×. **d** Western blotting relative to the expression of HERG1, TXNDC5, p21, cyclin D1, E-cadherin, vimentin, and fibronectin in the subcutaneous tumors (*n* = 3). **e** Representative bioluminescence imaging of nude mice and ex vivo imaging of the lungs and photographs and H&E staining of metastatic tumors in lung samples harvested on day 35 after caudal intravenous injection of ESCC cells from the three groups (*n* = 10). Magnification: 100×. *: *p* < 0.05

We then performed xenograft studies to investigate whether HERG1 deregulation is associated with tumorigenesis and/or metastasis formation. We subcutaneously injected KYSE-30 cells, transfecting scramble shRNA or two different shRNA targeting HERG1 (shHERG1 #1 and shHERG1 #2) into athymic female mice. All the injected mice formed tumors on their flanks, near their back legs (Fig. 7a). However, the growth indices of the shHERG1 #1 and shHERG1 #2 group tumors were substantially lower as compared to the growth index of tumors in the scrambled shRNA group (Fig. 7b). Histological examination validated this observation. Furthermore, consistent with these findings, we observed that the expression of Ki-67, a proliferation marker, significantly decreased upon HERG1 silencing (Fig. 7c). The reduction of the ESCC volume in the shHERG1 #1 and shHERG1 #2 mice was accompanied by downregulation of TXNDC5, cyclin D1, vimentin, and fibronectin expression and upregulation of p21 and E-cadherin expression (Fig. 7d).

Postmortem examinations performed on day 35 showed that fluorescence intensity was significantly decreased in mice and in the lungs of mice injected with shHERG1 #1 or shHERG1 #2 cells. Moreover, HERG1 knockdown reduced metastases in the lungs of each mouse, with histological examination confirming the presence of these pulmonary metastases (Fig. 7e).

Therefore, reduction of HERG1 expression effectively interfered with the potential of ESCC cells to proliferative and metastasize in vivo.

## Discussion

Despite many efforts, patients with ESCC continue to have a poor prognosis. To identify molecular markers capable of improving early diagnosis and accurate prognosis, we investigated the role of HERG1 in ESCC. We analyzed the expression and function of HERG1 in ESCC cells using different techniques, and found that HERG1 plays a role in ESCC progression and metastasis. Importantly, our data suggest targeting of HERG1 as a possible patient-tailored approach in the therapy of ESCC.

HERG1 has been proposed as a therapeutic target in cancer treatment [25]. Indeed, the expression of HERG1 is increased in different cancers, including gastric cancer, breast cancer, and pancreatic ductal adenocarcinoma [12–14]. Consistently, our results demonstrated that HERG1 is overall upregulated in hepatocellular carcinoma, bladder cancer, gastric cancer, and ovarian cancer, indicating that HERG1 upregulation is ubiquitous in various types of cancer. HERG1 is considered to play a critical role in the regulation of various cellular processes in tumor cells, such as proliferation, cell cycle, and angiogenesis [12, 13, 26]. However, the role of HERG1 in ESCC is still unclear. Our study is the first to demonstrate that HERG1 is overexpressed in both primary ESCC tumors and ESCC cell lines, whereas it is expressed at lower levels in the epithelial lining of normal esophageal mucosa. In addition, HERG1 upregulation is strongly associated with increased overall TNM stages and shorter survival times. Therefore, HERG1 might be used as a predictive marker for outcomes in patients with ESCC. Here, we observed that decreased HERG1 expression reduced the growth and metastasis of ESCC xenografts in nude mice, and negatively affected cell proliferation, cell cycle, migration, and invasion. These findings support the clinical results of our study and agree with the effects of HERG1 observed in other cancer types [12–15], thereby implying that HERG1 might promote ESCC progression.

EMT is critical for cancer metastasis; it involves the downregulation of epithelial markers and the upregulation of mesenchymal ones, with consequent loss of cell-cell contact and increased migratory capabilities of the cells [27, 28]. We found that changes in HERG1 levels altered the expression of EMT markers in ESCC cells, and that HERG1 levels are associated with EMT markers in ESCC tumor samples. Our results suggest that HERG1 induces a malignant phenotype in ESCC cells and makes them more motile and invasive by inducing EMT.

Aberrant TXNDC5 expression has been reported in multiple malignancies, and is involved in the modulation of tumor cell cycle, proliferation, and migration, likely representing an important event in the progression of cervical cancer, prostate cancer, and lung cancer [18, 19, 29]. However, the effects of TXNDC5 deregulation have not been studied in ESCC, yet, and this is the first study in this regard. Previous studies have shown that both HERG1 and TXNDC5 are involved in tumor cell proliferation, invasion, and angiogenesis, and may be useful in predicting the outcomes of certain tumor patients [12, 16, 18, 30–33]. Meanwhile, both HERG1 and TXNDC5 interact with the integrin β1 subunit at the plasma membrane and activate the NF-κB signaling pathway [15, 34–36]. Moreover, dysregulation of HERG1 or TXNDC5 may contribute to myocardial disease [7, 35]. The substantial commonality of HERG1 and TXNDC5 suggests that there may be a relationship between them. In the present study, we showed that, in patients, HERG1 expression was associated with TXNDC5 expression. In addition, a survival analysis revealed that the prognosis of patients who exhibited high TXNDC5 expression was clearly poorer than that of patients exhibiting medium or low TXNDC5 expression. Importantly, silencing or overexpression of HERG1 affected proliferation, invasion, and EMT in vitro, and these effects were reversed by overexpression or downregulation of TXNDC5, respectively, strongly indicating that TXNDC5 is critical for the function of HERG1. Our results are the first to show a mechanistic relationship between an ion channel-related protein and TXNDC5 and show that HERG1 exerts its effect on tumor progression by regulation of the oncogene TXNDC5 expression.

**Fig. 8:**
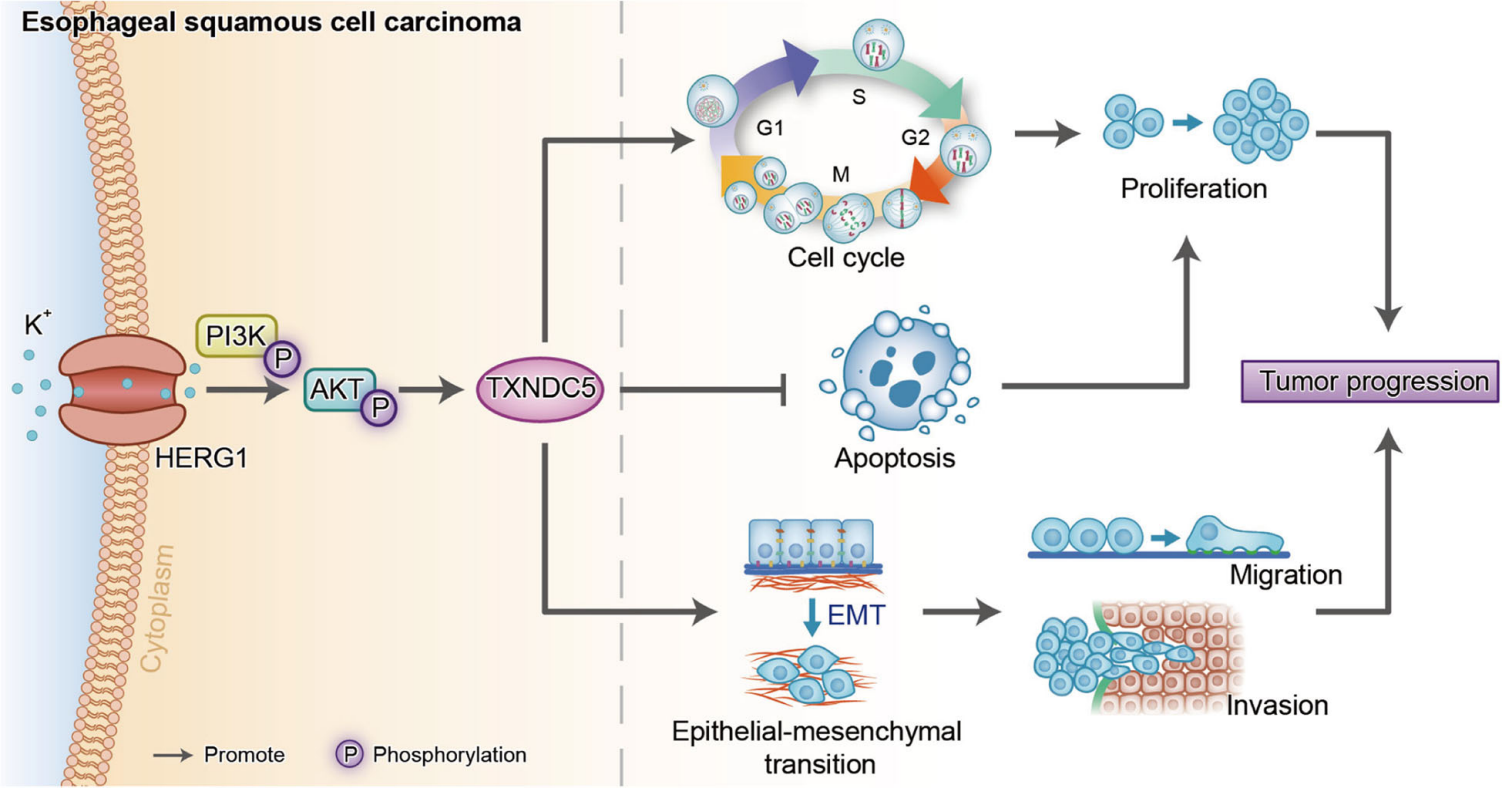
Diagram summarizing the role of HERG1 in promoting ESCC progression via TXNDC5. HERG1 promotes the phosphorylation of PI3K and AKT and, in turn, potentiates TXNDC5 expression, resulting in acceleration of the cell cycle process, inhibition of cell apoptosis, and induction of the EMT, consequently boosting ESCC cell proliferation, migration, and invasion, therefore affecting the prognosis of ESCC patients

The PI3K/AKT signaling pathway is involved in tumor progression in various types of cancer, including ESCC [37, 38]; however, its relationship with HERG1 and TXNDC5 remains unclear. Here, we investigated the involvement of the PI3K/AKT signaling pathway in HERG1-mediated TXNDC5 expression, which accelerates ESCC progression. Our findings suggested that this process relies, at least in part, upon TXNDC5, and that HERG1 expression represents an upstream element of PI3K/AKT signaling. The role of HERG1 in promoting ESCC progression is summarized in Fig. 8.

## Conclusions

In this study, we investigated the significance of HERG1 in ESCC and demonstrated that (1) HERG1 expression is significantly upregulated in ESCC; (2) HERG1 upregulation is associated with a higher overall TNM stage and a shorter survival time; (3) HERG1 augments proliferation, cell cycle, migration, invasion, and EMT of ESCC cells; (4) HERG1 inhibition impedes tumor growth and metastasis in vivo; (5) HERG1 regulates the expression of the oncogene TXNDC5, which is also related to the prognosis of patients with ESCC demonstrated in this study and affects the development of ESCC; and (6) the PI3K/AKT signaling pathway is involved in HERG1-mediated upregulation of TXNDC5. Based on these results, we speculate that HERG1 might be a novel therapeutic target for ESCC. However, further studies are needed to validate the impact of HERG1 on clinical course or response to chemotherapy or radiotherapy.

## Additional file

Additional file 1: **Figure S1.** F-actin appearance of ESCC cells in different treatment groups was observed by immunofluorescence staining. **Table S1.** Primer sequences for HERG1 and TXNDC5 overexpression. **Table S2.** Short hairpin RNA (shRNA) sequences for HERG1 silencing. **Table S3.** Short interfering RNA (siRNA) sequences targeting TXNDC5. **Table S4.** Primers used for quantitative polymerase chain reaction (qPCR) analyses. **Table S5.** Clinicopathological characteristics of patients with ESCC exhibiting high, medium, and low intensity HERG1 immunohistochemical staining. **Table S6.** Clinicopathological characteristics of patients with ESCC exhibiting high, medium, and low intensity TXNDC5 immunohistochemical staining. (DOC 573 kb)

## Data Availability

The datasets used and/or analyzed during the current study are available from the corresponding author upon reasonable request.

## Abbreviations

BrdU: Bromodeoxyuridine
BSA: Bovine serum albumin
DAPI: 4′,6-diamidino-2-phenylindole
EMT: Epithelial-mesenchymal transition
ESCC: Esophageal squamous cell carcinoma
FBS: fetal Bovine serum
GAPDH: Glyceraldehyde-3-phosphate dehydrogenase
H&E: Hematoxylin and eosin
HERG1: Human ether a-go-go related gene 1
IHC: Immunohistochemistry
JNK: Janus N-terminal kinase
M-MLV-RT: Moloney murine leukemia virus reverse transcriptase
MTT: Methyl thiazolyl tetrazolium
PBS: Phosphate-buffered saline
PI: Propidium iodide
PI3K: Phosphoinositide 3-kinase
qPCR: Quantitative polymerase chain reaction
RPMI: Roswell Park Memorial Institute medium
shRNA: Short hairpin RNA
siRNA: Small interfering RNA
TUNEL: Terminal deoxynucleotidyl transferase mediated dUTP-biotin nick end labeling
TXNDC5: Thioredoxin domain-containing protein 5
