# Supplementary material for: HERG1 promotes esophageal squamous cell carcinoma growth and metastasis through TXNDC5 by activating the PI3K/AKT pathway
Source: J Exp Clin Cancer Res. 2019 Jul 22;38:324. doi: 10.1186/s13046-019-1284-y (PMC6647263; doi:10.1186/s13046-019-1284-y)
Supplement: Supplementary file 1 — Figure S1. F-actin appearance of ESCC cells in different treatment groups was observed by immunofluorescence staining. Table S1. Primer sequences for HERG1 and TXNDC5 overexpression. Table S2. Short hairpin RNA (shRNA) sequences for HERG1 silencing. Table S3. Short interfering RNA (siRNA) sequences targeting TXNDC5. Table S4. Primers used for quantitative polymerase chain reaction (qPCR) analyses. Table S5. Clinicopathological characteristics of patients with ESCC exhibiting high, medium, and low intensity HERG1 immunohistochemical staining. Table S6. Clinicopathological characteristics of patients with ESCC exhibiting high, medium, and low intensity TXNDC5 immunohistochemical staining. (DOC 573 kb) [file 13046_2019_1284_MOESM1_ESM.doc]

**SUPPLEMENTARY INFORMATION**

**Supplementary Figure 1**

**
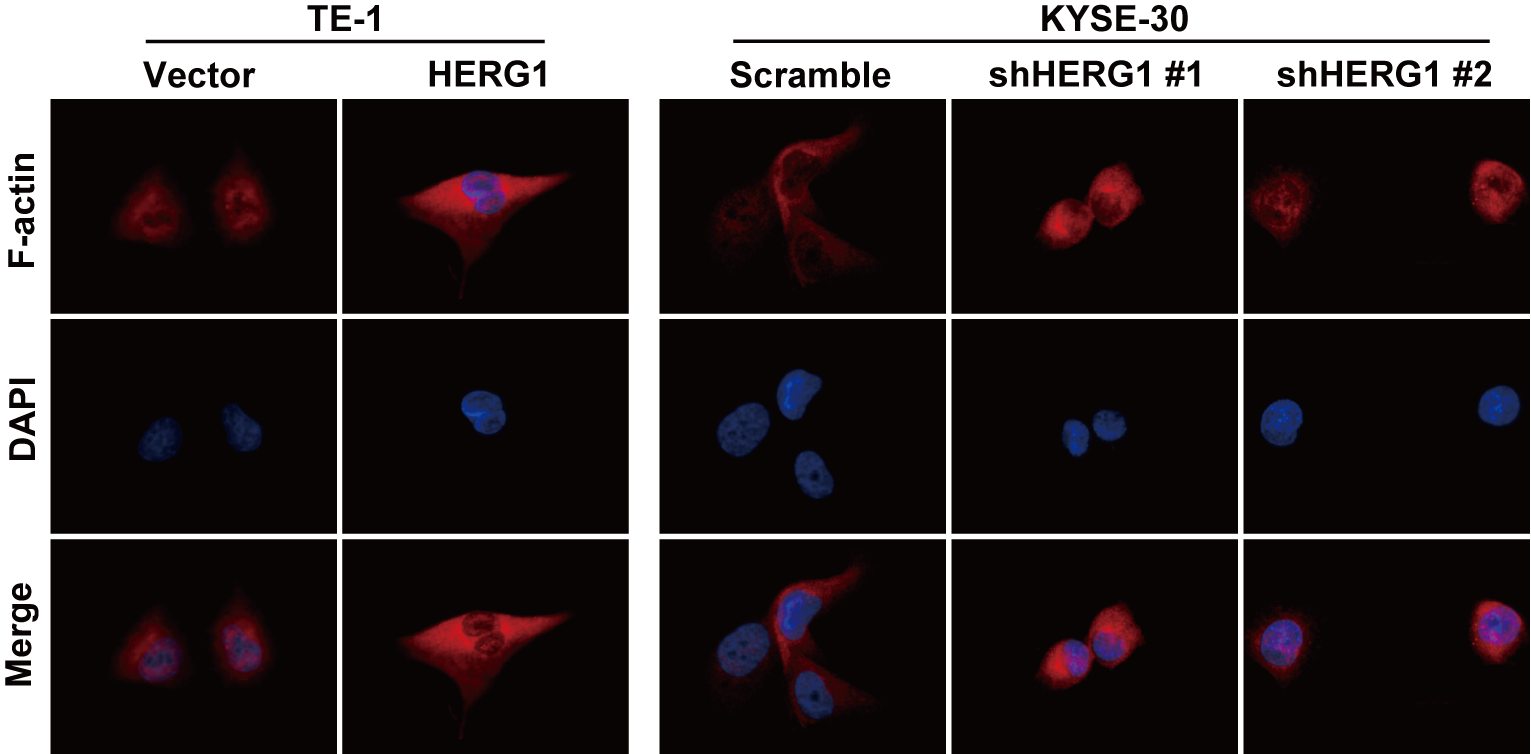
**

**Supplementary Figure 1. F-actin appearance of ESCC cells in different treatment groups was observed by immunofluorescence staining.**

**Supplementary Table** 1. Primer sequences for HERG1 and TXNDC5 overexpression

| **Gene name** | **Sequence (5´ to 3´)** |
| --- | --- |
| ***HERG1*** | FW: ATGCCCGCGCGCCCAGGACG  RV: CTAAAGTTCGTCTTTCGCTTG |
| ***TXNDC5*** | FW: ATGCCGGTGCGGAGGGGCCA  RV: CTAACTGCCCGGGTCCGAGCCG |

**Supplementary Table** 2. Short hairpin RNA (shRNA) sequences for HERG1 silencing

| **Gene name** | **Sequence (5´ to 3´)** |
| --- | --- |
| ***HERG1 (#1)*** | GTTCATCATCGCCAACGCT |
| ***HERG1 (#2)*** | GTGGAAATCGCCTTCTACC |

**Supplementary Table 3.** Short interfering RNA (siRNA) sequences targeting TXNDC5

| **Gene name** | **Sequence (5´ to 3´)** |
| --- | --- |
| ***TXNDC5*** | ATCGAGCTACTTCCCATAATA |

**Supplementary Table 4. Primers used for quantitative polymerase chain reaction (qPCR) analyses**

| **Gene name** | **Primer sequence (5´ to 3´)** |
| --- | --- |
| ***HERG1*** | FW: GTGGAAATCGCCTTCTACCG  RV: GCCCCATCCTCGTTCTTCA |
| ***E-cadherin*** | FW: ATGAGTGTCCCCCGGTATCT  RV: CAAACACGAGCAGAGAATCA |
| ***Vimentin*** | FW: CGCCAGATGCGTGAAATGG  RV: ACCAGAGGGAGTGAATCCAGA |
| ***Fibronectin*** | FW: ATGATGAGGTGCACGTGTGT  RV: CCCTGACCGAAGCATGTACA |
| ***p21*** | FW: GGGACAGCAGAGGAAGACC  RV: GACTAAGGCAGAAGATGTAGAGC |
| ***CyclineD1*** | FW: GGTGGCAAGAGTGTGGAG  RV: CCTGGAAGTCAACGGTAGC |
| ***TXNDC5*** | FW: CTCTGGGCCTTGAACATT  RV: CCCTCAGTGACTCCAAA |
| ***GAPDH*** | FW: GAAGGTGAAGGTCGGAGTC  RV: GAAGATGGTGATGGGATTTC |

**Supplementary Table 5. Clinicopathological characteristics of patients with ESCC exhibiting high, medium, and low intensity HERG1 immunohistochemical staining**

| **Characteristic** | ***n* (%)** | **HERG1 expression** | | | ***p*-value** |
| --- | --- | --- | --- | --- | --- |
|  |  | **High (*n*, %)** | **Medium (*n*, %)** | **Low (*n*, %)** |  |
| Gender | | | | | 0.968 |
| Male | 274 (78.5%) | 49 (17.9%) | 168 (61.3%) | 57 (20.8%) |  |
| Female | 75 (21.5%) | 12 (16.0%) | 49 (65.3%) | 14 (18.7%) |  |
| Age (years) | | | | | 0.131 |
| ≥ 60 | 245 (70.2%) | 38 (15.5%) | 154 (62.9%) | 53 (21.6%) |  |
| < 60 | 104 (29.8%) | 23 (22.1%) | 63 (60.6%) | 18 (17.3%) |  |
| TNM stage | | | |  | **0.000** |
| I | 30 (8.6%) | 2 (6.7%) | 15 (50.0%) | 13 (43.3%) |  |
| II | 120 (34.4%) | 9 (7.5%) | 75 (62.5%) | 36 (30.0%) |  |
| III | 154 (44.1%) | 28 (18.2%) | 106 (68.8%) | 20 (13.0%) |  |
| IV | 45 (12.9%) | 22 (48.9%) | 21 (46.7%) | 2 (4.4%) |  |
| T grade | | | |  | **0.006** |
| T1 | 24 (6.9%) | 3 (12.5%) | 13 (54.2%) | 8 (33.3%) |  |
| T2 | 79 (22.6%) | 9 (11.4%) | 51 (64.6%) | 19 (24.1%) |  |
| T3 | 237 (67.9%) | 44 (18.6%) | 149 (62.9%) | 44 (18.6%) |  |
| T4 | 9 (2.6%) | 5 (55.6%) | 4 (44.4%） | 0 (0.0%) |  |
| N grade | | | | | **0.000** |
| N0 | 141 (40.4%) | 10 (7.1%) | 86 (61.0%) | 45 (31.9%) |  |
| N1 | 103 (29.5%) | 13 (12.6%) | 73 (70.9%) | 17 (16.5%) |  |
| N2 | 68 (19.5%) | 20 (29.4%) | 41 (60.3%) | 7 (10.3%) |  |
| N3 | 37 (10.6%) | 18 (48.6%) | 17 (45.9%) | 2 (5.4%) |  |
| Survival | | | | | **0.000** |
| Yes | 80 (22.9%) | 4 (5.0%) | 50 (62.5%) | 26 (32.5%) |  |
| No | 269 (77.1%) | 57 (21.2%) | 167 (62.1%) | 45 (16.7%) |  |

**Supplementary Table 6. Clinicopathological characteristics of patients with ESCC exhibiting high, medium, and low intensity TXNDC5 immunohistochemical staining**

| **Characteristic** | ***n* (%)** | **TXNDC5 expression** | | | ***p*-value** |
| --- | --- | --- | --- | --- | --- |
|  |  | **High (*n*, %)** | **Medium (*n*, %)** | **Low (*n*, %)** |  |
| Gender | | | | | 0.482 |
| Male | 274 (78.5%) | 75 (27.4%) | 143 (52.2%) | 56 (20.4%) |  |
| Female | 75 (21.5%) | 16 (21.3%) | 44 (58.7%) | 15 (20.0%) |  |
| Age (years) | | | | | 0.747 |
| ≥ 60 | 245 (70.2%) | 65 (26.5%) | 127 (51.8%) | 53 (21.6%) |  |
| < 60 | 104 (29.8%) | 26 (25.0%) | 60 (57.7%) | 18 (17.3%) |  |
| TNM stage | | | |  | **0.000** |
| I | 30 (8.6%) | 1 (3.3%) | 15 (50.0%) | 14 (46.7%) |  |
| II | 120 (34.4%) | 21 (17.5%) | 63 (52.5%) | 36 (30.0%) |  |
| III | 154 (44.1%) | 45 (29.2%) | 89 (57.8%) | 20 (13.0%) |  |
| IV | 45 (12.9%) | 24 (53.3%) | 20 (44.4%) | 1 (2.2%) |  |
| T grade | | | |  | **0.009** |
| T1 | 24 (6.9%) | 2 (8.3%) | 15 (62.5%) | 7 (29.2%) |  |
| T2 | 79 (22.6%) | 22 (27.8%) | 36 (45.6%) | 21 (26.6%) |  |
| T3 | 237 (67.9%) | 61 (25.7%) | 133 (56.1%) | 43 (18.1%) |  |
| T4 | 9 (2.6%) | 6 (66.7%) | 3 (33.3%） | 0 (0.0%) |  |
| N grade | | | | | **0.000** |
| N0 | 141 (40.4%) | 21 (14.9%) | 73 (51.8%) | 47 (33.3%) |  |
| N1 | 103 (29.5%) | 24 (23.3%) | 61 (59.2%) | 18 (17.5%) |  |
| N2 | 68 (19.5%) | 26 (38.2%) | 37 (54.4%) | 5 (7.4%) |  |
| N3 | 37 (10.6%) | 20 (54.1%) | 16 (43.2%) | 1 (2.7%) |  |
| Survival | | | | | **0.000** |
| Yes | 80 (22.9%) | 4 (5.0%) | 43 (53.8%) | 33 (41.3%) |  |
| No | 269 (77.1%) | 87 (32.3%) | 144 (53.5%) | 38 (14.1%) |  |
